# Lightweight End-to-End Deep Learning Solution for Estimating the Respiration Rate from Photoplethysmogram Signal

**DOI:** 10.3390/bioengineering9100558

**Published:** 2022-10-16

**Authors:** Moajjem Hossain Chowdhury, Md Nazmul Islam Shuzan, Muhammad E. H. Chowdhury, Mamun Bin Ibne Reaz, Sakib Mahmud, Nasser Al Emadi, Mohamed Arselene Ayari, Sawal Hamid Md Ali, Ahmad Ashrif A. Bakar, Syed Mahfuzur Rahman, Amith Khandakar

**Affiliations:** 1 Department of Electrical, Electronic and System Engineering, Universiti Kebangsaan Malaysia, Bangi 43600, Malaysia; 2 Department of Electrical Engineering, Qatar University, Doha 2713, Qatar; 3 Department of Civil and Architectural Engineering, Qatar University, Doha 2713, Qatar; 4 Technology Innovation and Engineering Education Unit (TIEE), Qatar University, Doha 2713, Qatar; 5 Department of Biomedical Engineering, Military Institute of Science and Technology, Mirpur Cantonment, Dhaka 1216, Bangladesh

**Keywords:** photoplethysmogram, respiration rate, machine learning, deep learning, ConvMixer, convolutional neural networks

## Abstract

Respiratory ailments are a very serious health issue and can be life-threatening, especially for patients with COVID. Respiration rate (RR) is a very important vital health indicator for patients. Any abnormality in this metric indicates a deterioration in health. Hence, continuous monitoring of RR can act as an early indicator. Despite that, RR monitoring equipment is generally provided only to intensive care unit (ICU) patients. Recent studies have established the feasibility of using photoplethysmogram (PPG) signals to estimate RR. This paper proposes a deep-learning-based end-to-end solution for estimating RR directly from the PPG signal. The system was evaluated on two popular public datasets: VORTAL and BIDMC. A lightweight model, ConvMixer, outperformed all of the other deep neural networks. The model provided a root mean squared error (RMSE), mean absolute error (MAE), and correlation coefficient (R) of 1.75 breaths per minute (bpm), 1.27 bpm, and 0.92, respectively, for VORTAL, while these metrics were 1.20 bpm, 0.77 bpm, and 0.92, respectively, for BIDMC. The authors also showed how fine-tuning a small subset could increase the performance of the model in the case of an out-of-distribution dataset. In the fine-tuning experiments, the models produced an average R of 0.81. Hence, this lightweight model can be deployed to mobile devices for real-time monitoring of patients.

## 1. Introduction

Heart rate, blood pressure, body temperature, and respiration rate (RR) are considered the four primary vital signs for diagnosing any abnormality in the human body. RR is defined as the number of breaths taken by a person in a minute (breaths/minute). Any sudden spike or fall in RR is often seen as a sign of the body malfunctioning [1,2,3]. An increase in RR can strongly indicate problems with the respiratory system [4], cardiac arrest [5], and rapid collapse of the body resulting in death [6]. Furthermore, RR can be used to identify pneumonia [7,8], pulmonary embolism [9,10], hypercarbia [11], and sepsis. It is adopted by emergency departments in hospitals as a screening parameter [12]. It can also be used as an early detector for COVID-19, as some research studies [13,14] have shown. As a result, it can be concluded that RR should be monitored by healthcare personnel to diagnose any acute decline in a patient’s health [15]. Thus, it is logical that patients have their RR measured regularly. This is usually done after every few hours [16].

In light of these use cases, RR must be calculated regularly and accurately by healthcare workers. Unfortunately, the common way of doing this is by manually counting the number of breaths taken by the patients. This is cumbersome and is not possible when long-term monitoring of a patient is required. On top of that, it introduces human error in estimating RR [17,18]. A way of tackling this problem is to use capnography. This method measures the concentration of partial pressure of carbon dioxide in respiratory gases [19]. This method is very accurate, but the device is unwieldy in usage. Hence, this method is primarily used in the case of anesthesia or when patients are in intensive care. Keeping all of these factors in mind, it is thus important that other alternative methods for estimating RR should be developed.

Current research is focused on using either electrocardiogram (ECG) or photoplethysmogram (PPG) for the estimation of RR. These signals are very rich in information and have been used for many other applications ranging from blood pressure estimation to even user authentication [20,21,22,23]. The motivation for using these signals for alternative RR is twofold. Firstly, with the advent of wearable devices, ECG and PPG can be easily acquired [24] and can be used by non-healthcare professionals. Secondly, these signals allow for non-invasive RR estimation. As a result, the patient will face less discomfort and will have a lower chance of becoming infected. There are many methods [25,26,27] for estimating RR from ECG that reported good accuracy. However, in a study [28], it has been observed that, in some ICU patients, the respiratory signals extracted from ECG were not accurate, despite the patients still breathing. This is a major concern. Hence PPG signals are seen as the more viable approach for estimating RR.

The recent developments in estimating RR from ECG and PPG are reviewed thoroughly in [29,30,31]. In [30,31], more than 100 algorithms have been proposed for the estimation of RR from PPG and ECG. The central theme is to extract a representation of the respiratory signal and then estimate RR from it. The authors in [7] put forward a new method of estimating RR from PPG. They derived three respiratory-induced variations (frequency, period, and amplitude) from PPG. The estimations from these variations were fused in a process called smart fusion to obtain a better approximation of RR. They achieved a root mean square error (RMSE) of 3.0 breaths per minute (bpm), which was better than the RR estimation from individual respiratory-induced variations. Another study [32] investigated new algorithms for estimating RR from children in the emergency department. Segments of PPG signals that were contaminated by motion artifacts were removed automatically. Their algorithm was performed with a mean absolute error (MAE) of 5.2 bpm.

Zhang et al. [33] introduced an algorithm that uses joint sparse signal reconstruction and spectra fusion to estimate RR from PPG. Another study [34] investigated the use of amplitude variability of finger PPG and compared it to the approach of using four time–frequency signal representations cascaded with a particle filter to estimate RR. In [35], ten subjects following intensive care unit (ICU) discharge were studied. RR was estimated from PPG signals and accelerometry data. In [36], the authors investigated the difference in RR estimated from PPG at various body sites for different breathing conditions. RR was estimated via spectral power density from 36 healthy subjects. In [37], the authors estimated the RR of 201 patients in the ICU. Respiration-induced frequency components were used for estimating RR. Ensemble empirical mode decomposition (EEMD) was used to estimate RR on two different datasets in [38]. A smart fusion method based on ensemble empirical mode decomposition was used to improve the estimation of RR from PPG [39]. Rathore et al. [40] used a U-net model with residual-inception blocks to synthesize a respiration signal from which they estimated RR. They used a very deep model with six layers for this task, which makes it non-suitable for portable devices. Lampier et al. [41] used deep neural networks that include convolution and long short-term memory (LSTM) layers to estimate RR from PPG.

The different RR estimation algorithms from PPG are summarized in Supplementary Table S1. Very little work has been performed using ML or deep learning in estimating RR from PPG. With the availability of large, annotated datasets [29,42], it is viable to use deep learning to estimate RR. In our previous study [43], we used feature extraction and classical machine learning to estimate RR. The major motivation behind this study is to use more sophisticated technologies, such as deep neural networks, to estimate RR robustly.

The major contributions of this work are as follows:

- a lightweight deep neural network for estimating RR, which will enable deployment in various devices;
- evaluation of the model in both intra-dataset and inter-dataset settings to ensure generalization capabilities;
- the ability of the deep learning model to estimate the RR of an out-of-distribution dataset by fine-tuning a small subset;
- robust error analysis of the results to ensure the reliability of the models.

This paper is divided into four sections. Section 1 provides an overview of the use of PPG in RR estimation as well as a summary of the current research work in this domain. Section 2 describes the dataset used, preprocessing steps, the models trained, as well as the training methodology. Section 3 shows the results from the various experiments and discusses the implication of the results. The performance of this work is then compared to the current state-of-the-art methods in the same section. Section 4 wraps up the whole paper as a conclusion.

## 2. Materials and Methods

**Figure 1:**
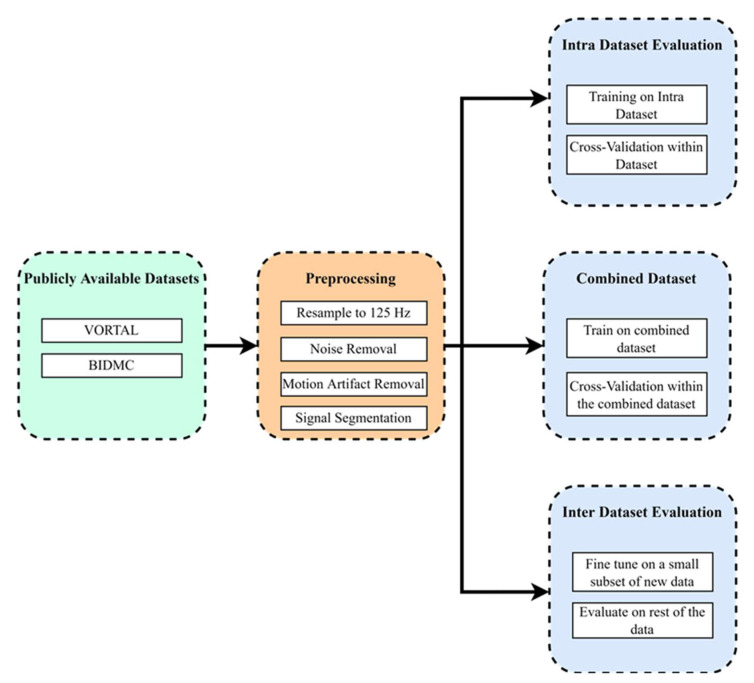
Overview of the methodology of this work.

In this section, the methodology of this work is discussed. Two publicly available datasets are considered in this study. The datasets are first preprocessed before any model training. The signals are resampled, denoised, and segmented into smaller windows. The preprocessed data are then used to train deep learning models. A cross-validation scheme is used to train and evaluate the models. The overall process is depicted in Figure 1, and the processes are explained in this section.

### 2.1. Preprocessing

The signals of both datasets are first resampled to a fixed sample rate so that the model can be trained and evaluated on both datasets. In normal conditions, it is very common to have motion artifacts (MAs) and high-frequency noise in the acquired PPG signal. The motion artifacts can range from spikes in data to distortion of the signal’s fiducial points. These corruptions will obstruct the deep neural networks from learning meaningful features from the signals. To rectify that, a low-pass filter is used to remove the noise. A low-pass Butterworth infinite impulse response (IIR) zero-phase filter [44] was implemented in MATLAB. The filter was of the sixth order and had a cut-off frequency of 25 Hz. Supplementary Figure S1 depicts the effect of the low-pass filter. The blue color line represents the raw signal, and the orange color line represents the filtered data. Supplementary Figure S1a shows a 16 s segment of the signal, but it is difficult to see the effect properly. In Supplementary Figure S1b, a zoomed-in version of 2 s is shown. It can be seen that some of the high-frequency noise is removed owing to the low pass filter.

To remove motion artifact (MA) from the signal, variational mode decomposition (VMD) [45,46] has been found to be robust and quite effective [43]. In [43], it has been shown that the last mode out of the five modes extracted from a PPG signal contains most of the MA. The same configuration was used in this work as well. The signals are then segmented to 16 s windows with an overlap of 50%. This is done because it is very difficult for a deep learning model to work on a very large signal segment.

### 2.2. Neural Network Architectures

In this work, five neural network architectures were considered: ResNet [47], DenseNet [48], Inception_v1 [49], MobileNet [50], and ConvMixer [51]. These networks were proposed for two-dimensional (2D) problems or image domain problems. As an image can be thought of as a 2D signal, these networks were adapted for this 1D problem of estimating the RR.

**ResNet:** ResNet is a type of neural network that introduces the concept of skip connections. A ResNet model usually contains a multiple-layer skip connection with nonlinearities and batch normalization in between. The idea behind skip connection is to avoid the problem of vanishing gradients in deeper models.

**DenseNet:** In DenseNet, within a dense block, the output of each layer is connected to the output of every other layer. In other words, for each layer, the outputs of previous layers are considered separate inputs, and their own output is passed as an input for the next layers. A DenseNet consists of multiple such dense blocks.

**Inception_v1:** Inception_v1 or GoogleNet introduced the concept of concatenating convolution layers with different kernel sizes. This is because it allows the model to “view” the data from different perspectives.

**MobileNet:** MobileNets were proposed as neural network architectures that were able to perform usual deep learning tasks with very low parameter counts. As a result, they are ideal for use cases that require low latency and low power. This architecture uses depthwise separable convolutions, which significantly reduces the number of parameters when compared with the network with normal convolutions with the same depth in the networks. The normal convolution is replaced by depthwise convolution, followed by pointwise convolution, which is called depthwise separable convolution.

**Figure 2:**
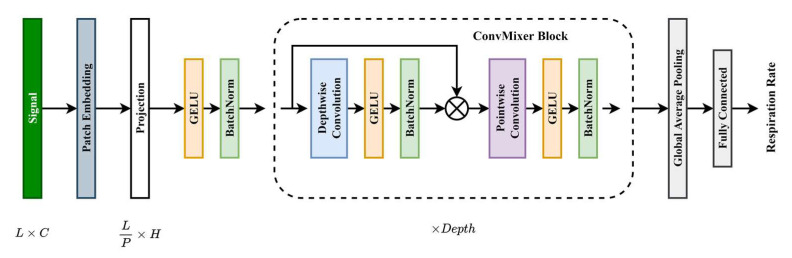
ConvMixer architecture.

**ConvMixer:** The ConvMixer architecture was proposed to investigate whether patches are the reason for improved performance in vision tasks. Hence, the first layer of ConvMixer is a patch embedding layer. This is achieved with the help of a convolution layer, where the kernel size and stride are equal to the patch size. This converts a signal with *L* length and *C* channels to a projection of *L*/*P* length and *H* channels, where *P* refers to the patch size. This is followed by a nonlinearity and a batch normalization layer. The nonlinearity or activation layer used is the Gaussian error linear unit or GELU. The second part of the model is a ConvMixer block. This block consists of a residual block containing depthwise convolution, an activation layer, and a batchnorm layer. The inputs are concatenated with the output of the batchnorm layer. The concatenated output is then followed by pointwise convolution, an activation layer, and a batchnorm layer. The ConvMixer block is repeated *Depth* times. The final part of the model contains a global average pooling layer and a fully connected layer. RR is then calculated using linear activation. The network architecture is shown in Figure 2.

### 2.3. Dataset Description

Two datasets were used in this study: VORTAL [29] and BIDMC [42]. Both datasets contain PPG, ECG, and ground truth RR. The BIDMC dataset is a subset of the MIMIC-II dataset [52], where data were collected from ICU patients. BIDMC contains data from 53 subjects. The VORTAL dataset contains data from 39 subjects. The PPG signals were acquired in resting conditions. The datasets are summarized in Table 1.

The signals from the dataset were segmented into windows of 16 seconds with 50% overlap. This ensures that there is enough time for breaths to take place without sacrificing the number of samples for training the deep learning models. The PPG signals from VORTAL were resampled to 125 Hz to maintain parity with BIDMC. Here, 2981 and 2980 signals were collected from VORTAL and BIDMC, respectively. 

### 2.4. Training Methodology

The neural networks described in this work were implemented using Tensorflow and Keras. The networks were trained for 500 epochs with a batch size of 128. Early stopping criteria were introduced to prevent overfitting by stopping the training if the validation loss diverged for more than 50 epochs. Mean squared error loss was minimized in this experiment. An Adam optimizer with a learning rate of 1 × 10^−3^ was used to optimize the networks. The models were evaluated using fivefold cross-validation. That is to say, for each fold, 20% of data were reserved for testing, and 80% were reserved for “training + validation”. The “training + validation” data were then split further into 80% and 20% for the training set and validation set, respectively.

### 2.5. Evaluation Criteria

Five evaluation criteria were utilized in this investigation. Here, *X_p_* indicates the projected data, *X* is the ground truth data, and n denotes the number of samples or recordings.

- Mean absolute error (MAE): MAE is the average of the absolute errors. This is one of the standard metrics for regression problems.
  (1) $$\mathrm{MAE}=\frac{1}{n}\sum_{n}\left|X_{p}-X\right|$$
- RMSE (root mean squared error): RMSE is the square root of the mean of squared errors. This metric is very harsh when the predictions and ground truth differ largely.
  (2) $$\mathrm{RMSE}=\sqrt{\frac{\sum {\left|X_{p}-X\right|}^{2}}{n}}$$
- Correlation coefficient (R): R is used to calculate the degree to which two variables (prediction and ground truth) are linked. This is a scale-invariant metric that allows for reliable comparison between multiple datasets.
  (3) $$R=\sqrt{1-\frac{MSE\left(Model\right)}{MSE\left(Baseline\right)}}$$
  where MSE (baseline) = $\frac{\sum {\left|X-mean\left(X\right)\right|}^{2}}{n}$
- 2SD: Standard deviation (SD) is a statistical technique that measures the spread of data relative to its mean. 2SD is significant as it indicates the 95% confidence interval.
  (4) $$2\mathrm{SD}=1.96\;\times \;\mathrm{SD}=1.96\;\sqrt{\frac{\sum error-mean{\left(error\right)}^{2}}{n}}$$
  where error = $X_{p}-X$
- Limit of agreement (LOA): LOA allows for errors resulting from random and systematic events. Hence, it is helpful to assess the reliability of the predictions of the models. In this work, 95% LOAs were calculated.

In this study, R was prioritized as the main metric for evaluation. A paired sample *ttest* was conducted to find if the performance of a specific model is significant compared with the other models.

## 3. Results and Discussion

This section contains the numerical results of the experiments and the implication behind the results. The intra-dataset results are first discussed, then some possible inter-dataset evaluation settings are investigated, and the results are compared with the recent works published.

### 3.1. Intra Dataset Evaluation

#### 3.1.1. VORTAL

Five models were trained on the VORTAL dataset. Table 2 shows the fivefold cross-validation results on the dataset. It can be seen that ConvMixer significantly outperforms the other models (Supplementary Figure S2). Furthermore, the model also has the fewest parameters compared with other models. Hence, this model is used for further investigation. The ConvMixer model has a kernel size of 7, a patch size of 10, a channel of 256, and a depth of 8.

**Figure 3:**
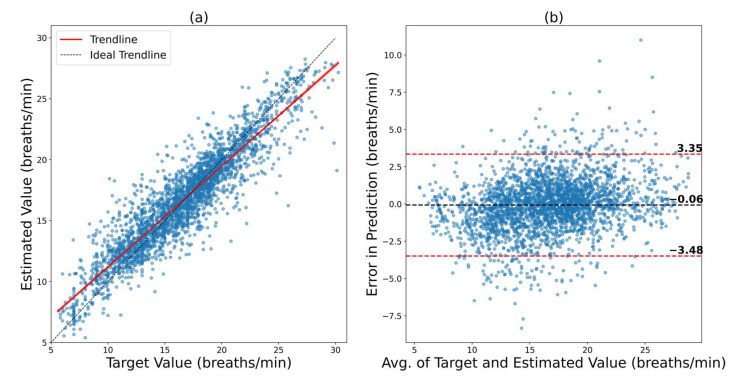
Results of ConvMixer on the VORTAL dataset. (**a**) Regression plot that shows how close the trendline is to the ideal trendline. (**b**) Bland–Altman plot that shows the 95% confidence interval of the error.

In Figure 3, we can see the results of ConvMixer visualized on the Vortal dataset. Figure 3a shows the regression plot where the predictions are plotted against the ground truth. It can be seen that most of the data are clustered along the ideal trendline (*y = x* line). This suggests that the model has a high correlation, which is verified by the R-value of 0.92. The trendline of the scatterplot is also very close to the ideal trendline. In Figure 3b, the Bland–Altman plot is depicted. The Bland–Altman plot shows the spread of the error and the 95% confidence interval (CI) of the error. The 95% CI is from −3.48 bpm to 3.35 bpm. This shows that the predictive ability of the model within the VORTAL dataset is remarkable.

#### 3.1.2. BIDMC

**Figure 4:**
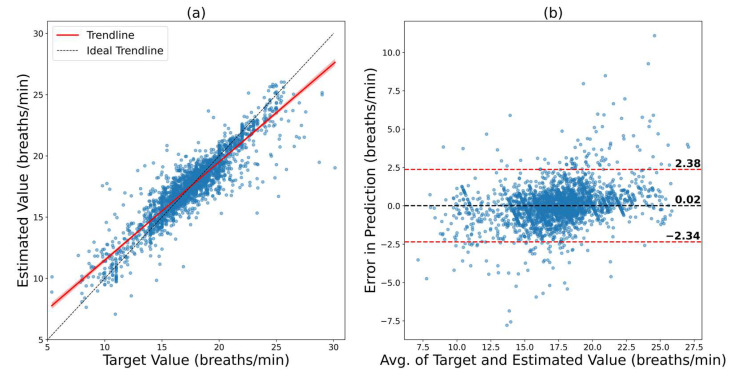
Results of ConvMixer on the BIDMC dataset. (**a**) Regression plot that shows how close the trendline is to the ideal trendline. (**b**) Bland–Altman plot that shows the 95% confidence interval of the error.

As ConvMixer was the best performer on the VORTAL dataset, this model was used to train on BIDMC data from scratch. Other models were also trained for BIDMC, and their results are summarized in Supplementary Table S2. All of the hyperparameters for ConvMixer were the same as before. The model’s predictions had an RMSE of 1.2039 bpm and an MAE of 0.7656 bpm. The correlation between the ground truth and the predictions (R) was 0.9155. The results are visualized in Figure 4. It can be seen that, in the regression plot, the trendline is very close to the ideal trendline (as proved by the R of 0.9155), and the 95% CI in the Bland–Altman plot is from −2.34 bpm to 2.38 bpm. The model has performed very well in BIDMC as well.

### 3.2. Inter Dataset Evaluation

#### 3.2.1. Combined Dataset

To test the robustness of the models, the model trained on BIDMC was tested on VORTAL and vice versa. The results were not good. The model trained on BIDMC, when tested on VORTAL, gave an RMSE of 4.98 bpm. On the other hand, the model trained on VORTAL and tested on BIDMC gave an RMSE of 5.78 bpm. These are poor prediction performances when compared with the intra-dataset performance. This makes some sense as the VORTAL dataset consists of healthy patients, while BIDMC consists of ICU patients. The difference in their physiology is likely making one dataset out of the distribution of the other.

**Figure 5:**
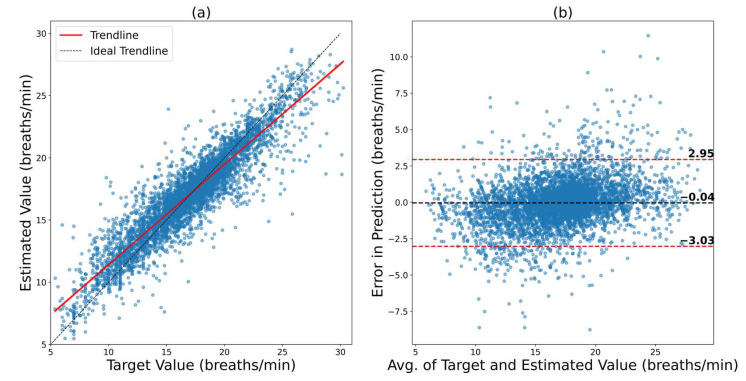
Results of ConvMixer on the combined dataset using fivefold cross-validation. (**a**) Regression plot that shows how close the trendline is to the ideal trendline. (**b**) Bland–Altman plot that shows the 95% confidence interval of the error.

To investigate whether this is the case, both datasets were combined and were trained and tested in a fivefold cross-validation scheme. The training hyperparameters were the same as those for individual dataset training. The results for the combined dataset are depicted in Figure 5. In Figure 5a, it can be observed that the trendline is very close to the ideal trendline, which results in a correlation coefficient of 0.9183 between the ground truth and predictions. The Bland–Altman plot in Figure 5b shows an LOA from 2.95 bpm to −3.03 bpm. This means that the errors are within a very small range. The RMSE and MAE for this scenario were 1.5246 bpm and 1.0417 bpm, respectively. This shows a massive boost in performance. Hence, when possible, the dataset in training should always have a good spread of different types of subjects.

To further study the robustness of the neural network in an inter-dataset setting, fine-tuning of the models were studied.

#### 3.2.2. Fine-Tuning on a Small Subset of the New Dataset

**Figure 6:**
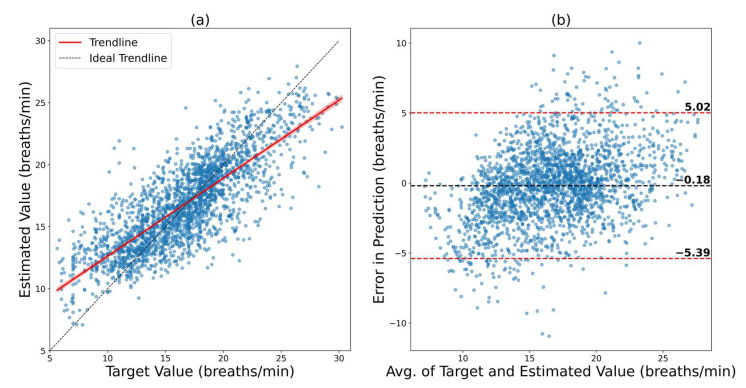
Results of ConvMixer, which was trained on BIDMC and later fine-tuned on the VORTAL dataset. (**a**) Regression plot that shows how close the trendline is to the ideal trendline. (**b**) Bland–Altman plot that shows the 95% confidence interval of the error.

In this case, the model trained on the BIDMC dataset was fine-tuned on a small sample of the VORTAL dataset. Here, 10% of the available data were used for training (fine-tuning), and another 10% were used for validation. The remaining 80% were used for testing. The results of this scenario are depicted in Figure 6. The regression plot shows a decent agreement between the ground truth and prediction, with an R of 0.8017. The Bland–Altman plot shows an LOA from 5.02 bpm to −5.39 bpm. The model had an RMSE and MAE of 2.6609 bpm and 2.0174 bpm, respectively. This shows a dramatic improvement from the scenario where the BIDMC model was tested without fine-tuning (RMSE improved from 4.98 bpm to 2.66 bpm). The scenario was repeated where the model was trained on VORTAL and fine-tuned on BIDMC to verify if this method works in the reverse scenario as well.

**Figure 7:**
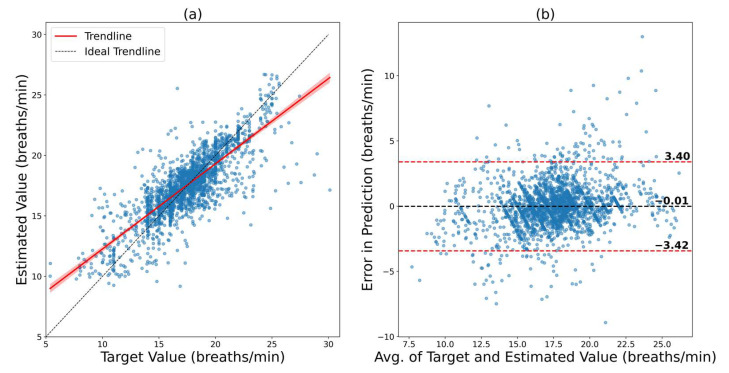
Results of ConvMixer, which was trained on VORTAL and later fine-tuned on the BIDMC dataset. (**a**) Regression plot that shows how close the trendline is to the ideal trendline. (**b**) Bland–Altman plot that shows the 95% confidence interval of the error.

In this case, the model trained on the VORTAL dataset was fine-tuned on a small sample of the BIDMC dataset. Here, 10% of the available data were used for training (fine-tuning), and another 10% were used for validation. The remaining 80% were used for testing. The results of this scenario are depicted in Figure 7. The regression plot shows a decent agreement between the ground truth and prediction, with an R of 0.8123. The Bland–Altman plot shows an LOA from 3.40 bpm to −3.42 bpm. The model had an RMSE and MAE of 1.7403 bpm and 1.1838 bpm, respectively. This again shows a remarkable improvement in prediction capability (RMSE improves from 5.78 bpm to 1.74 bpm). Hence, there is another method of improving the performance of the model in an out-of-distribution dataset by fine-tuning it on a small subsample of the new dataset.

### 3.3. Comparison with Literature

The overall results of this work are summarized in Table 3. The intra-dataset evaluation in this work has shown remarkable results in predicting out-of-fold samples for both datasets. The models gave an R of 0.9209 and 0.9155 for VORTAL and BIDMC, respectively. Combining both of the datasets to include more variation in the training set allowed us to train models that had an R of 0.9183. Fine-tuning on small subsets of BIDMC and VORTAL gave an R of 0.8123 and 0.8017, respectively. This metric shows that the models trained in this work can be used for new scenarios with just a few samples for fine-tuning.

The recent research work in estimating RR from PPG is summarized in Table 4 and is compared with this work in terms of the details of the dataset used, methods applied, and the result reported. There are several practical limitations when comparing work with the performance reported in the literature. Owing to the presence of data from a diverse group of subjects, lack of consistent criteria for evaluating performance, and absence of uniform algorithm implementations, it is difficult to make a like-for-like comparison. Thus, in this work, we have evaluated our model as fairly as possible and used multiple metrics. Pirhonen et al. [34] proposed a method of using amplitude variations of PPG signals to predict RR. In that study, the VORTAL dataset was used along with wavelet synchro-squeezing transform to estimate RR with an MAE and RMSE of 2.33 bpm and 3.68 bpm, respectively. Shuzan et al. [43] used machine learning models along with feature extraction to estimate RR from the VORTAL dataset. Their best result came from using the best features selected by a fitted Gaussian process regression (GPR) model. They achieved RMSE, MAE, and 2SD of 2.63 bpm, 1.97 bpm, and 5.25 bpm, respectively. Our models that were evaluated on VORTAL outperform those in the literature with an RMSE of 1.75 bpm (intra-dataset), beating the RMSE of 2.63 bpm of Shuzan et al. The inter-dataset result of 2.66 bpm is also very close to Shuzan et al.’s result.

Jarchi et al. [35] used only 10 subjects from BIDMC to estimate RR from PPG signals relative to the accelerometer with an MAE of 2.56 bpm. Lampier et al. [41] extracted respiratory-induced intensity variation, respiratory-induced amplitude variation, and respiratory-induced frequency variation signals from PPG. These signals were then fed to a deep neural network to estimate RR. The BIDMC dataset was used to obtain an MAE of 3.4 bpm. Our models that were evaluated on BIDMC outperformed the reported results in the literature (on BIDMC). Our models achieved an MAE of 0.77 bpm (intra-dataset) and 1.18 bpm (inter-dataset), outperforming Jarchi et al.’s result of 2.56 bpm.

The low error in the prediction of RR is a major advantage of our work over signal processing methods. Furthermore, it can be seen that the performance of this work has low variation in error, which means that the model is robust over multiple samples. Deep learning models are also found to be more robust to unusual signals compared with signal processing methods. In clinical practices, it is often the need for an accurate and robust monitoring system that is crucial. In terms of deployability, the lightweight nature of this model ensures that it is not hampered by the need for heavy hardware.

There is no established medical standard for estimating RR. Despite that, a review study [29] where the authors studied 196 signal processing algorithms for RR estimation was carried out. The authors claimed that an MAE of less than 2 bpm suggests a good estimator. In Table 3, most of the results have an MAE of less than 2 bpm, while only the ‘BIDMC model fine-tuned on VORTAL’ has an MAE of 2.02 bpm. The difference is very small, so it can be stated that all of the models pass this criterion.

## 4. Conclusions

In this study, the authors proposed the ConvMixer architecture for estimating RR from PPG signals. The authors leveraged previously established preprocessing techniques and ConvMixer to achieve state-of-the-art results.

The models were evaluated in both intra-dataset and inter-dataset configurations. By combining both datasets, the authors achieved a very high correlation coefficient between the predictions and ground truth, thus confirming that, in the case of RR estimation, the diversity of the training set is very important. When it is not feasible to combine such large datasets, fine-tuning on a small subset produces acceptable results. The authors showed that fine-tuning on just 10% of a dataset allows the model to improve the result dramatically. In the intra-dataset configuration, the models, on average, achieved a correlation coefficient of 0.92 between the predictions and ground truth. Furthermore, with just 0.56 million parameters, the model is very light and hence suitable for deployment in mobile devices. This state-of-the-art performance of the proposed system will ensure that the system will work accurately when deployed and can be used for wearable remote RR monitoring applications.

## Figures and Tables

**Table 1:** Dataset summary of VORTAL and BIDMC.

		Median	Range
*VORTAL*	**Sex (female)**	54%	-
	**Age (years)**	29	18–39
	**BMI (kg/m^2^)**	23	-
	**RR (bpm)**	-	5–32
	**PPG Sample Rate (Hz)**	500	-
*BIDMC*	**Sex (female)**	60%	-
	**Age (years)**	-	19–90+
	**RR (bpm)**	-	5–25
	**PPG Sample Rate (Hz)**	125	-

**Table 2:** Evaluation of five convolution neural networks using fivefold cross-validation.

Model	Parameters (Millions)	R	MAE (bpm)	RMSE (bpm)
Resnet18	0.93	0.6462	2.6926	3.4274
Inception_v1	3.40	0.8239	1.8698	2.5463
Mobilenet_v1	2.01	0.7349	2.4252	3.1651
Densenet121	277.36	0.7494	2.2265	2.9825
**ConvMixer**	**0.56**	**0.9209**	**1.2702**	**1.7450**

**Table 3:** Summary of the investigations in this work.

Scenario	R	RMSE (bpm)	MAE (bpm)	LOA (bpm)	2SD (bpm)
*Fivefold cross-validation on VORTAL*	0.9209	1.7450	1.2702	−3.48 to 3.35	3.42
*Fivefold cross-validation on BIDMC*	0.9155	1.2039	0.7656	−2.34 to 2.38	2.36
*Fivefold cross-validation on the combined dataset*	0.9183	1.5246	1.0417	−3.03 to 2.95	2.99
*BIDMC model fine-tuned on VORTAL*	0.8017	2.6609	2.0174	−5.39 to 5.02	5.21
*VORTAL model fine-tuned on BIDMC*	0.8123	1.7403	1.1838	−3.42 to 3.40	3.41

**Table 4:** Summary of recent related works with this work, including the database, methodology, and estimation error for RR. MAE, RMSE, and 2SD are in bpm.

Author	Database	Subject	Method	Metric	Result
***Pirhonen*et al.** [34]	Vortal	39	Wavelet Synchro squeezing Transform	MAE RMSE R 2SD	2.33 3.68 - -
***Jarchi*et al.** [35]	BIDMC	10	Accelerometer	MAE RMSE R 2SD	2.56 - - -
***Shuzan*et al.** [43]	Vortal	39	Machine Learning	MAE RMSE R 2SD	1. 97 2.63 0.88 5.25
***Lampier*et al.** [41]	BIDMC	53	Deep Neural Network	MAE RMSE R 2SD	3.4 6.9 - -
**This work** **(Intra Dataset)**	Vortal	39	**ConvMixer**	MAE RMSE R 2SD	**1.27** **1.75** **0.92** **3.42**
**This work** **(Intra Dataset)**	BIDMC	53	**ConvMixer**	MAE RMSE R 2SD	**0.77** **1.20** **0.92** **2.36**

## Data Availability

This work used publicly available datasets collected from [29,42].

## Supplementary Materials

The following supporting information can be downloaded at https://www.mdpi.com/article/10.3390/bioengineering9100558/s1, Figure S1: Applying a low pass filter to remove high-frequency noises. (a) Effect of filter on the whole signal (b) Zooming on 2 s data to see the effect of low pass filter, Figure S2: Paired sample ttest results showing that ConvMixer outperformed other models significantly, Table S1: Summary of the current research in estimating RR, Table S2: Evaluation of Five Convolution Neural Networks using 5-fold cross validation on BIDMC dataset.

## References

[1] Fieselmann J.F., Hendryx M.S., Helms C.M., Wakefield D.S. (1993). Respiratory Rate Predicts Cardiopulmonary Arrest for Internal Medicine Inpatients. J. Gen. Intern. Med..

[2] Goldhill D.R., White S.A., Sumner A. (1999). Physiological Values and Procedures in the 24 h before ICU Admission from the Ward. Anaesthesia.

[3] Ebell M.H. (2007). Predicting Pneumonia in Adults with Respiratory Illness. Am. Fam. Physician.

[4] Gravelyn T.R., Weg J.G. (1980). Respiratory Rate as an Indicator of Acute Respiratory Dysfunction. JAMA.

[5] Schein R.M.H., Hazday N., Pena M., Ruben B.H., Sprung C.L. (1990). Clinical Antecedents to In-Hospital Cardiopulmonary Arrest. Chest.

[6] Duckitt R.W., Buxton-Thomas R., Walker J., Cheek E., Bewick V., Venn R., Forni L.G. (2007). Worthing Physiological Scoring System: Derivation and Validation of a Physiological Early-Warning System for Medical Admissions. An Observational, Population-Based Single-Centre Study. Br. J. Anaesth..

[7] Karlen W., Raman S., Ansermino J.M., Dumont G.A. (2013). Multiparameter Respiratory Rate Estimation from the Photoplethysmogram. IEEE Trans. Biomed. Eng..

[8] Khalil A., Kelen G., Rothman R.E. (2007). A Simple Screening Tool for Identification of Community-Acquired Pneumonia in an Inner City Emergency Department. Emerg. Med. J..

[9] Pimentel M.A.F., Charlton P.H., Clifton D.A. (2015). Probabilistic Estimation of Respiratory Rate from Wearable Sensors. Wearable Electronics Sensors.

[10] Goldhaber S.Z., Visani L., De Rosa M. (1999). Acute Pulmonary Embolism: Clinical Outcomes in the International Cooperative Pulmonary Embolism Registry (ICOPER). Lancet.

[11] Cretikos M.A., Bellomo R., Hillman K., Chen J., Finfer S., Flabouris A. (2008). Respiratory Rate: The Neglected Vital Sign. Med. J. Aust..

[12] Farrohknia N., Castrén M., Ehrenberg A., Lind L., Oredsson S., Jonsson H., Asplund K., Göransson K.E. (2011). Emergency Department Triage Scales and Their Components: A Systematic Review of the Scientific Evidence. Scand. J. Trauma. Resusc. Emerg. Med..

[13] Miller D.J., Capodilupo J.V., Lastella M., Sargent C., Roach G.D., Lee V.H., Capodilupo E.R. (2020). Analyzing Changes in Respiratory Rate to Predict the Risk of COVID-19 Infection. PLoS ONE.

[14] Rahman T., Akinbi A., Chowdhury M.E.H., Rashid T.A., Sengür A., Khandakar A., Islam K.R., Ismael A.M. (2022). COV-ECGNET: COVID-19 Detection Using ECG Trace Images with Deep Convolutional Neural Network. Heal. Inf. Sci. Syst..

[15] Cretikos M., Chen J., Hillman K., Bellomo R., Finfer S., Flabouris A., Investigators M.S. (2007). The Objective Medical Emergency Team Activation Criteria: A Case--Control Study. Resuscitation.

[16] William B., Albert G., Ball C., Bell D., Binks R., Durham L., Eddleston J., Edwards N., Evans D., Jones M. (2012). National Early Warning Score (NEWS): Standardizing the Assessment of Acute Illness Severity in the NHS. Rep. Work. Party.

[17] Lovett P.B., Buchwald J.M., Stürmann K., Bijur P. (2005). The Vexatious Vital: Neither Clinical Measurements by Nurses nor an Electronic Monitor Provides Accurate Measurements of Respiratory Rate in Triage. Ann. Emerg. Med..

[18] Philip K.E.J., Pack E., Cambiano V., Rollmann H., Weil S., O’Beirne J. (2015). The Accuracy of Respiratory Rate Assessment by Doctors in a London Teaching Hospital: A Cross-Sectional Study. J. Clin. Monit. Comput..

[19] Jaffe M.B. (2008). Infrared Measurement of Carbon Dioxide in the Human Breath:“Breathe-through” Devices from Tyndall to the Present Day. Anesth. Analg..

[20] Chowdhury M.H., Shuzan M.N.I., Chowdhury M.E.H., Mahbub Z.B., Uddin M.M., Khandakar A., Reaz M.B.I. (2020). Estimating Blood Pressure from the Photoplethysmogram Signal and Demographic Features Using Machine Learning Techniques. Sensors.

[21] Chowdhury M.E.H., Alzoubi K., Khandakar A., Khallifa R., Abouhasera R., Koubaa S., Ahmed R., Hasan A. (2019). Wearable Real-Time Heart Attack Detection and Warning System to Reduce Road Accidents. Sensors.

[22] Ibtehaz N., Chowdhury M.E.H., Khandakar A., Kiranyaz S., Rahman M.S., Tahir A., Qiblawey Y., Rahman T. (2021). EDITH: ECG Biometrics Aided by Deep Learning for Reliable Individual AuTHentication. IEEE Trans. Emerg. Top. Comput. Intell..

[23] Shen Y., Voisin M., Aliamiri A., Avati A., Hannun A., Ng A. Ambulatory Atrial Fibrillation Monitoring Using Wearable Photoplethysmography with Deep Learning. Proceedings of the 25th ACM SIGKDD International Conference on Knowledge Discovery & Data Mining.

[24] Adochiei N.I., David V., Tudosa I. (2011). Methods of Electromagnetic Interference Reduction in Electrocardiographic Signal Acquisition. Environ. Eng. Manag. J..

[25] Moody G.B., Mark R.G., Zoccola A., Mantero S. (1985). Derivation of Respiratory Signals from Multi-Lead ECGs. Comput. Cardiol..

[26] Orphanidou C., Fleming S., Shah S.A., Tarassenko L. (2013). Data Fusion for Estimating Respiratory Rate from a Single-Lead ECG. Biomed. Signal Process. Control.

[27] Mirmohamadsadeghi L., Vesin J.-M. (2014). Respiratory Rate Estimation from the ECG Using an Instantaneous Frequency Tracking Algorithm. Biomed. Signal Process. Control.

[28] Drew B.J., Harris P., Zègre-Hemsey J.K., Mammone T., Schindler D., Salas-Boni R., Bai Y., Tinoco A., Ding Q., Hu X. (2014). Insights into the Problem of Alarm Fatigue with Physiologic Monitor Devices: A Comprehensive Observational Study of Consecutive Intensive Care Unit Patients. PLoS ONE.

[29] Charlton P.H., Birrenkott D.A., Bonnici T., Pimentel M.A.F., Johnson A.E.W., Alastruey J., Tarassenko L., Watkinson P.J., Beale R., Clifton D.A. (2017). Breathing Rate Estimation from the Electrocardiogram and Photoplethysmogram: A Review. IEEE Rev. Biomed. Eng..

[30] Charlton P.H., Bonnici T., Tarassenko L., Clifton D.A., Beale R., Watkinson P.J. (2016). An Assessment of Algorithms to Estimate Respiratory Rate from the Electrocardiogram and Photoplethysmogram. Physiol. Meas..

[31] Charlton P.H., Bonnici T., Tarassenko L., Alastruey J., Clifton D.A., Beale R., Watkinson P.J. (2017). Extraction of Respiratory Signals from the Electrocardiogram and Photoplethysmogram: Technical and Physiological Determinants. Physiol. Meas..

[32] Shah S.A., Fleming S., Thompson M., Tarassenko L. (2015). Respiratory Rate Estimation during Triage of Children in Hospitals. J. Med. Eng. Technol..

[33] Zhang X., Ding Q. (2017). Respiratory Rate Estimation from the Photoplethysmogram via Joint Sparse Signal Reconstruction and Spectra Fusion. Biomed. Signal Process. Control.

[34] Pirhonen M., Peltokangas M., Vehkaoja A. (2018). Acquiring Respiration Rate from Photoplethysmographic Signal by Recursive Bayesian Tracking of Intrinsic Modes in Time-Frequency Spectra. Sensors.

[35] Jarchi D., Rodgers S.J., Tarassenko L., Clifton D.A. (2018). Accelerometry-Based Estimation of Respiratory Rate for Post-Intensive Care Patient Monitoring. IEEE Sens. J..

[36] Hartmann V., Liu H., Chen F., Hong W., Hughes S., Zheng D. (2019). Towards Accurate Extraction of Respiratory Frequency from the Photoplethysmogram: Effect of Measurement Site. Front. Physiol..

[37] L’Her E., N’Guyen Q.-T., Pateau V., Bodenes L., Lellouche F. (2019). Photoplethysmographic Determination of the Respiratory Rate in Acutely Ill Patients: Validation of a New Algorithm and Implementation into a Biomedical Device. Ann. Intensive Care.

[38] Motin M.A., Karmakar C.K., Palaniswami M. (2019). Selection of Empirical Mode Decomposition Techniques for Extracting Breathing Rate from PPG. IEEE Signal Process. Lett..

[39] Motin M.A., Kumar Karmakar C., Kumar D.K., Palaniswami M. (2020). PPG Derived Respiratory Rate Estimation in Daily Living Conditions. Proc. Annu. Int. Conf. IEEE Eng. Med. Biol. Soc. EMBS.

[40] Rathore K.S., Vijayarangan S., SP P., Sivaprakasam M. (2021). A Deep Learning Based Multitask Network for Respiration Rate Estimation--A Practical Perspective. arXiv.

[41] Lampier L.C., Coelho Y.L., Caldeira E.M.O., Bastos-Filho T.F. (2022). A Deep Learning Approach to Estimate the Respiratory Rate from Photoplethysmogram. Ingenius.

[42] Pimentel M.A.F., Johnson A.E.W., Charlton P.H., Birrenkott D., Watkinson P.J., Tarassenko L., Clifton D.A. (2016). Toward a Robust Estimation of Respiratory Rate from Pulse Oximeters. IEEE Trans. Biomed. Eng..

[43] Shuzan M.N.I., Chowdhury M.H., Hossain M.S., Chowdhury M.E.H., Reaz M.B.I., Uddin M.M., Khandakar A., Bin Mahbub Z., Ali S.H.M. (2021). A Novel Non-Invasive Estimation of Respiration Rate from Motion Corrupted Photoplethysmograph Signal Using Machine Learning Model. IEEE Access.

[44] Chatterjee A., Roy U.K. (2018). PPG Based Heart Rate Algorithm Improvement with Butterworth IIR Filter and Savitzky-Golay FIR Filter. Proceedings of the 2018 2nd International Conference on Electronics, Materials Engineering & Nano-Technology (IEMENTech).

[45] Wang Y., Markert R. (2016). Filter Bank Property of Variational Mode Decomposition and Its Applications. Signal Processing.

[46] Roy B., Gupta R., Chandra J.K. (2019). Estimation of Respiration Rate from Motion Corrupted Photoplethysmogram: A Combined Time and Frequency Domain Approach. Proceedings of the 2019 IEEE Region 10 Symposium (TENSYMP).

[47] He K., Zhang X., Ren S., Sun J. (2016). Deep Residual Learning for Image Recognition. Proceedings of the IEEE Conference on Computer Vision and Pattern Recognition.

[48] Huang G., Liu Z., Van Der Maaten L., Weinberger K.Q. (2017). Densely Connected Convolutional Networks. Proceedings of the IEEE Conference on Computer Vision and Pattern Recognition.

[49] Szegedy C., Liu W., Jia Y., Sermanet P., Reed S., Anguelov D., Erhan D., Vanhoucke V., Rabinovich A. (2015). Going Deeper with Convolutions. Proceedings of the IEEE Conference on Computer Vision and Pattern Recognition.

[50] Howard A.G., Zhu M., Chen B., Kalenichenko D., Wang W., Weyand T., Andreetto M., Adam H. (2017). Mobilenets: Efficient Convolutional Neural Networks for Mobile Vision Applications. arXiv.

[51] Trockman A., Kolter J.Z. (2022). Patches Are All You Need?. arXiv.

[52] Saeed M., Villarroel M., Reisner A.T., Clifford G., Lehman L.-W., Moody G., Heldt T., Kyaw T.H., Moody B., Mark R.G. (2011). Multiparameter Intelligent Monitoring in Intensive Care II (MIMIC-II): A Public-Access Intensive Care Unit Database. Crit. Care Med..

