# Supplementary material for: Lightweight End-to-End Deep Learning Solution for Estimating the Respiration Rate from Photoplethysmogram Signal
Source: Bioengineering (Basel). 2022 Oct 16;9(10):558. doi: 10.3390/bioengineering9100558 (PMC9598342; doi:10.3390/bioengineering9100558)
Supplement: Supplementary file 1 [file bioengineering-09-00558-s001.zip › bioengineering-1936629-supplementary.pdf]

Supplementary Materials

Supplementary Table S1: Summary of the current research in estimating RR

Author	Method Used
Nilsson et al. [32]	Digital Filtering
Zhou et al. [33]	Independent Component Analysis
Fleming et al. [34]	Auto-regressive Model
Karlen et al. [7]	Fast Fourier Transformation
Orphanidou et al. [25]	Auto-regressive Model
Madhav et al. [35]	Modified multi scale principal component analysis
Mirmohamadsadegh et al. [26]	Instantaneous Frequency Tracking Algorithm
Philip et al. [18]	Spot Assessment
Pimentel et al. [9,36]	Auto-regressive Model
Zhang et al. [37,38]	Joint Sparse Signal Reconstruction
Lin et al. [39]	Wavelet-Based Algorithm
Moreno et al. [40]	Digital Filtering
Pirhonen et al. [41]	Wavelet-Based
Jarchi et al. [42]	Accelerometer Based
Hartmann et al. [43]	Fast Fourier Transformation
Motin et al. [44,45]	Ensemble Empirical Mode Decomposition
Shuzan et al. [46]	Classical Machine Learning
Lampier et al. [47]	Deep Learning
Rathore et al. [48]	Deep Learning

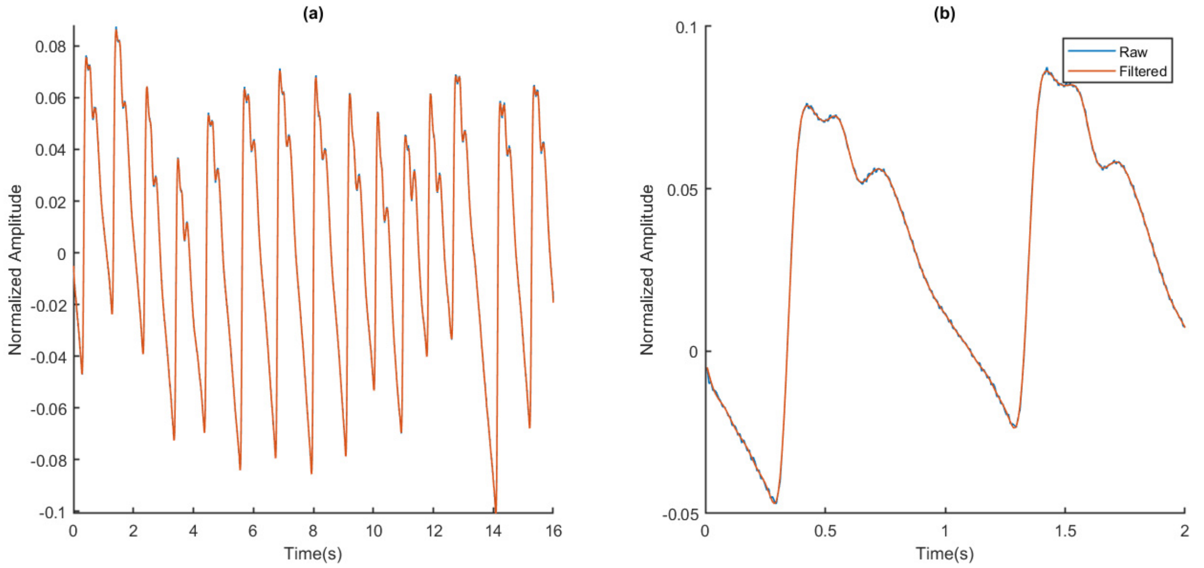

Supplementary Figure S1: Applying a low pass filter to remove high-frequency noises. (a) Effect of filter on the whole signal (b) Zooming on 2s data to see the effect of low pass filter

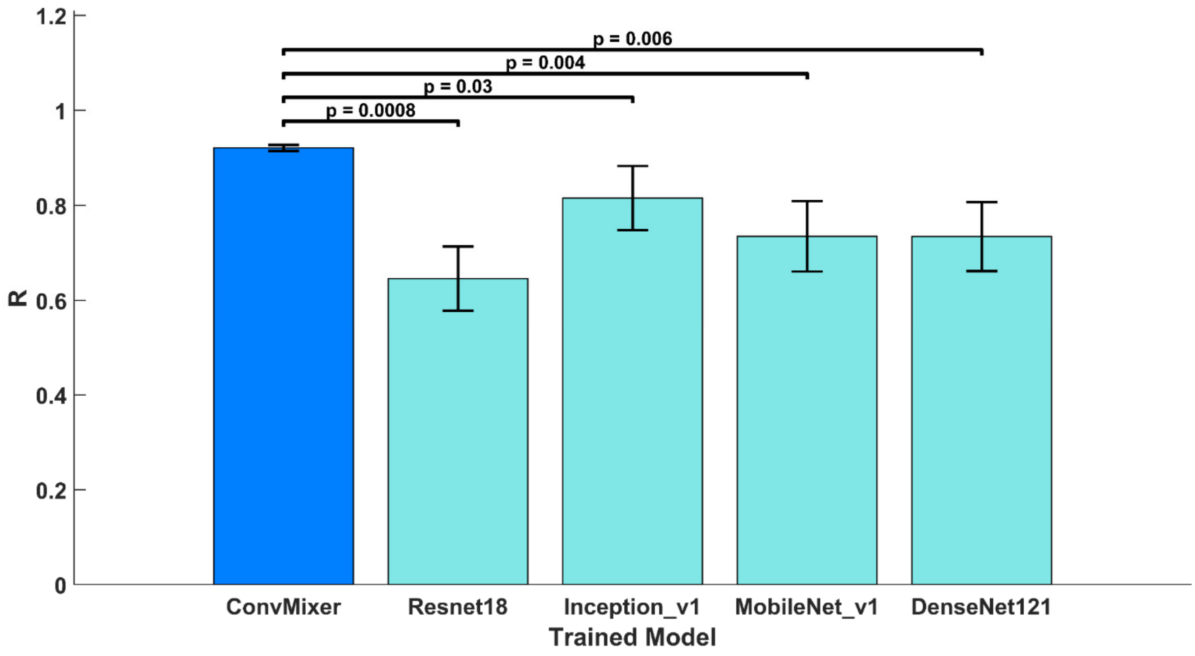

Supplementary Figure S2: Paired sample *ttest* results showing that ConvMixer outperformed other models significantly ($p<0.05$).

Supplementary Table S2: Evaluation of Five Convolution Neural Networks using 5-fold cross validation on BIDMC dataset

Model	Parameters (millions)	R	MAE (bpm)	RMSE (bpm)
Resnet18	0.93	0.7835	1.3186	1.8709
Inception_v1	3.40	0.7957	1.3535	1.8924
Mobilenet_v1	2.01	0.7818	1.3510	1.8930
Densenet121	277.36	0.8298	1.1219	1.6652
**ConvMixer**	**0.56**	**0.9155**	**0.7656**	**1.2039**
